# 晚期*ALK*融合阳性非小细胞肺癌治疗全程管理1例

**DOI:** 10.3779/j.issn.1009-3419.2021.101.32

**Published:** 2021-08-20

**Authors:** 赫男 白, 雨 韩, 意 牛, 明晖 张

**Affiliations:** 024000 赤峰，赤峰市医院肿瘤内三科 Department of Oncology, Chifeng City Hospital, Chifeng 024000, China

**Keywords:** 间变性淋巴瘤激酶, 克唑替尼, 恩沙替尼, 靶向治疗, 肺肿瘤, Anaplastic lymphoma kinase, Crizotinib, Ensartinib, Targeted therapy, Lung neoplasms

## Abstract

**背景与目的:**

间变性淋巴瘤激酶(anaplastic lymphoma kinase, *ALK*)是晚期非小细胞肺癌(non-small cell lung cancer, NSCLC)的重要治疗靶点。近几年来，随着多个ALK酪氨酸酶抑制剂(tyrosine kinase inhibitor, TKI)的出现，*ALK*融合阳性患者的总生存期(overall survival, OS)也在逐渐延长。现报道1例晚期*ALK*融合阳性NSCLC患者5余年的治疗经过，分析其治疗过程及效果评价，为后续患者的治疗提供经验。

**方法:**

回顾性分析内蒙古赤峰市医院肿瘤内科三病区2015年7月3日收治的1例晚期*ALK*融合突变阳性肺癌患者的诊疗过程。

**结果:**

患者男性，42岁，因“间断咳嗽、胸闷2个月，确诊肺腺癌1天”于2015年7月3日入我科。影像学检查提示左肺下叶占位，合并纵隔淋巴结肿大及左侧包裹性胸腔积液。支气管镜病理提示非小细胞癌，考虑腺癌。诊断：左肺下叶腺癌T1bN2M1a IV期。荧光原位杂交技术(fluorescence *in situ* hybridization, FISH)提示*ALK*(2p23)染色体易位。给予多西他赛+顺铂方案化疗2个周期，病情进展，改为培美曲塞+卡铂方案联合化疗6个周期，后续应用培美曲塞单药维持4个周期，疗效评估：部分缓解(partial remission, PR)。2016年4月9日给予克唑替尼治疗，2019年8月发现颅内多发转移，给予全脑放疗。2019年9月4日始口服恩沙替尼治疗至今。截至2021年3月1日，患者随访良好。

**结论:**

该晚期*ALK*融合阳性肺腺癌患者，一线及二线选用化疗，后线应用ALK-TKI治疗，总OS达68个月，目前随访良好。

## Abstract (en)

**Background and objective:**

Anaplastic lymphoma kinase (*ALK*) is an important therapeutic target for advanced non-small cell lung cancer (NSCLC). In recent years, with the emergence of several ALK tyrosine kinase inhibitors (TKI), the overall survival (OS) of *ALK* fusion positive patients is gradually extended. This paper reports the treatment of a late stage non-small cell lung cancer (NSCLC) patient with *ALK* fusion positive for more than 5 years, and analyzes the treatment process and effect evaluation, so as to provide experience for the follow-up treatment of patients.

**Methods:**

The diagnosis and treatment process of a patient with advanced *ALK* fusion mutation positive lung cancer admitted to the third ward of Department of oncology, Chifeng hospital, Inner Mongolia on July 3, 2015 was retrospectively analyzed.

**Results:**

A 42 years old male patient was admitted to our department on July 3, 2015 for "intermittent cough, chest tightness for 2 months, diagnosed with lung adenocarcinoma for 1 day". Imaging examination showed a space occupying lesion in the left lower lobe of the lung, accompanied by mediastinal lymphadenopathy and left encapsulated pleural effusion. Bronchoscopic pathology showed non-small cell carcinoma, and adenocarcinoma was tentatively suggested. Diagnosis: left lower lobe adenocarcinoma T1bN2M1a stage IV. Fluorescence *in situ* hybridization (FISH) indicated the translocation of *ALK* (2p23) chromosome. After 2 cycles of docetaxel+cisplatin (DP) regimens chemotherapy, disease progression occurred, so we used 6 cycles of pemetrexed+carboplatin to apply combination chemotherapy, 4 cycles of pemetrexed monotherapy were used after that. The efficacy evaluation: PR. On April 9, 2016, the patient was treated with crizotinib. In August 2019, multiple intracranial metastases were found and whole brain radiotherapy was given. Since September 4, 2019, oral administration of nsatinib has been carried out. As of March 1, 2021, the patients were followed up well.

**Conclusion:**

The advanced *ALK* fusion positive lung adenocarcinoma patients, though the first-line and the second-line chemotherapy, and the follwing application of ALK-TKI treatment, has procured a total OS has reached 68 months, and the current follow-up is good.

## 1 病例资料

患者男性，42岁，体表面积1.66 m^2^。因“间断咳嗽、胸闷2个月，确诊肺腺癌1天”于2015年7月3日就诊于我科。2015年5月患者无明显诱因出现间断咳嗽，伴胸闷、气短，时有心悸，无咯血，无胸痛，于当地诊所抗炎治疗无好转(具体不详)，2015年6月18日于外院行胸部计算机断层扫描(computed tomography, CT)提示：左肺炎症病变并局部肺组织膨胀不全，纵隔淋巴结肿大，左侧胸腔积液，心包积液。2015年6月19日行胸部增强CT：左肺下叶占位，较大层面大小2.9 cm×2.3 cm，考虑肺癌并阻塞性炎症，纵膈及左肺门多发淋巴结转移，左侧气胸及左侧包裹性积液。心脏彩超：心包少量积液。后患者就诊于北京大学肿瘤医院，行支气管镜检查提示左肺下叶癌，左主支气管隆起型病变。2015年7月2日病理回报：(左下叶基底段活检1块)非小细胞癌，考虑腺癌。既往否认结核病史、传染病及其他病史等。否认吸烟、饮酒史。查体：左肺呼吸音减弱，未闻及明显干湿啰音。初步诊断：左肺下叶腺癌T1bN2M1a IV期，体力状况(performance status, PS)评分1分。

入院后腹部增强CT及头部增强磁共振成像(magnetic resonance imaging, MRI)未见转移征象，胸腔彩超提示左侧胸腔积液，深约11.6 cm。评估无化疗禁忌，2015年7月8日始于我科行多西他赛+顺铂方案化疗2周期(多西他寒100 mg、d1;顺铂120 mg，分3天输注; *q3w*)，并予胸腔穿刺引流，胸水中查到肿瘤细胞，给予重组人血管内皮抑制素胸腔内注射配合胸部热疗控制积液。2015年7月15日基因检测回报(中国医学科学院肿瘤医院)：未检测到表皮生长因子受体(epidermal growth factor receptor, *EGFR*)第18、19、20、21号外显子突变; 未显示*KRAS*基因第2号外显子12、13密码子突变。荧光原位杂交技术(fluorescence *in situ* hybridization, FISH)结果显示：*ALK*(2p23)染色体易位。患者因经济原因拒绝调整为克唑替尼治疗。化疗2个周期后评估疗效：胸部及上腹部增强CT提示胸腔积液增多，心包积液，左肺下叶原发病灶较前增大(2.9 cm×2.3 cm→3.8 cm×3.4 cm)，纵隔淋巴结肿大，左侧肾上腺新发病灶，考虑转移。疗效评估：疾病进展(progressive disease, PD)。2015年8月19日调整为培美曲塞+卡铂方案化疗(培美曲塞800 mg、d1;卡铂500 mg、d2;*q3w*)。2个周期后评估疗效：胸闷、咳嗽症状好转，胸部及上腹部增强CT提示左下肺实性占位较前略小(3.8 cm×3.4 cm→3.4 cm×3.0 cm)，左侧包裹性胸腔积液较前减少，纵隔淋巴结肿大，左侧肾上腺实性占位未见明显改变。疗效评估：疾病稳定(stable disease, SD)。继续原方案化疗4个周期，自觉胸闷、咳嗽症状继续好转，6个周期后于2016年1月4日评效，复查胸部及上腹部增强CT提示肺部病灶较前缩小(3.4 cm×3.0 cm→2.8 cm×1.9 cm)，右侧胸腔积液及心包积液消失，左侧胸腔积液减少，纵隔淋巴结肿大，左侧肾上腺占位未见明显改变。疗效评估：SD。2016年1月5日继续培美曲塞二钠单药维持化疗3个周期(培美曲塞二钠800 mg、d1，*q3w*)。于2016年3月24日复查胸部及上腹部增强CT提示肺部病灶较前缩小(2.8 cm×1.9 cm→2.6 cm×1.4 cm)，左侧胸腔积液少量包裹性积液，纵隔淋巴结肿大，左侧肾上腺占位未见明显改变。疗效评估：部分缓解(partial remission, PR)(图 1)。继续培美曲塞单药维持1个周期，无进展生存期(progression-free survival, PFS)超过9个月。

**图 1:**
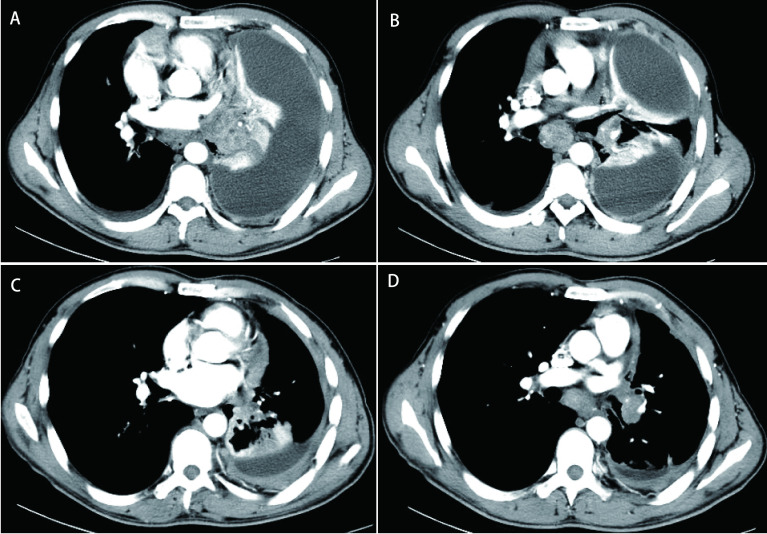
含培美曲塞方案化疗前及9个周期治疗后CT图像。A：化疗前CT图像(2015年8月17日)：左侧包裹性胸腔积液; B：化疗前CT图像(2015年8月17日)：纵隔淋巴结肿大，左肺下叶占位(3.8 cm×3.4 cm); C：9个周期化疗后CT图像(2016年3月24日)：左侧包裹性胸腔积液减少; D：9个周期化疗后CT图像(2016年3月24日)：纵膈肿大淋巴结缩小，左肺下叶占位缩小(2.6 cm×1.4 cm)。 CT images before and after 9 cycles of pemetrexed chemotherapy. A: CT images before chemotherapy (17-Aug-2015): left enveloped pleural effusion; B: CT images before chemotherapy (17-Aug-2015): mediastinal lymph node enlargement, left inferior lobe occupying space (3.8 cm×3.4 cm); C: CT images after 9 cycles of chemotherapy (24-Mar-2016): left enveloping pleural effusion was reduced; D: CT images after 9 cycles of chemotherapy (24-Mar-2016): mediastinal enlarged lymph node was reduced, left lower lobe area of the lung was reduced (2.6 cm×1.4 cm). CT: computed tomography.

经与患者充分沟通，2016年4月9日开始口服克唑替尼靶向治疗(250 mg, *bid*)，治疗第1周，出现“恶心、呕吐”症状，常见不良反应事件评价标准(common terminology criteria for adverse events, CTCAE)2级，调整给药时间，由空腹给药改为餐后给药，嘱患者进食清淡、易消化食物，少量多餐，少吃甜食及易产气食物，1周后消化道反应耐受。治疗第4周化验提示肝酶水平增高，CTCAE 1级，给予保肝治疗，至第8周，肝酶水平升高达CTCAE 3级，诊断克唑替尼相关性药物性肝损伤，给予停药，加用注射用还原型谷胱甘肽及注射用甘草酸二胺静脉输注治疗。治疗第9周肝酶水平降至CTCAE 2级，继续口服克唑替尼治疗，剂量不变。此后患者治疗过程顺利。治疗期间定期复查，2016年6月15日复查胸及上腹部增强CT，提示肺部病灶较前缩小(2.6 cm×1.4 cm→1.8 cm×0.7 cm)，左侧胸腔积液减少，纵隔淋巴结缩小，左侧肾上腺占位略缩小。疗效评估：PR。至2019年6月25日复查胸及上腹部平扫CT，提示左肺部病灶较前缩小(1.3 cm×0.6 cm)，左侧胸腔积液、纵隔淋巴结肿大较前好转，左侧肾上腺占位无明显变化。疗效评估：维持PR(图 2)。2019年8月初患者出现头晕，逐渐加重，并出现双下肢乏力、活动不灵，2019年8月6日于我院门诊复查头部MRI(图 3)提示：颅内多发异常信号，转移瘤可能性大(转移病灶大于3处)。疗效评估：PD(PFS为40个月)。2019年8月10日-2019年8月30日行头部放疗，具体：CT定位后行适行放疗，照射全脑30 Gy/2.5 Gy/12 f，局部病灶加量15 Gy/3 Gy/5 f。期间给予脱水降颅压治疗。同时行二次基因检测，因肿瘤组织取材困难，完善血液检测，提示未见*EGFR*、*ALK*、*KRAS*、*ROS1*、*MET*、*ERBB2*、*BRAF*、*RET*基因突变或融合。2019年9月4日始口服三代ALK-TKI恩沙替尼治疗(225 mg, po, *qd*)。治疗第2周化验提示肝酶水平增高，CTCAE 1级，加用注射用还原型谷胱甘肽联合注射用甘草酸二胺静脉输注治疗。治疗第4周患者转氨酶降至正常。后续患者服用恩莎替尼治疗过程顺利。治疗期间定期评估疗效，患者头晕、双下肢活动不灵症状缓解，2020年1月20日复查头MRI(图 3)及胸、腹部增强CT，疗效评估：SD。至2020年8月12日，疗效评估：SD(图 4)。截至2021年3月1日，仍维持恩沙替尼治疗，PFS超过18个月。

**图 2:**
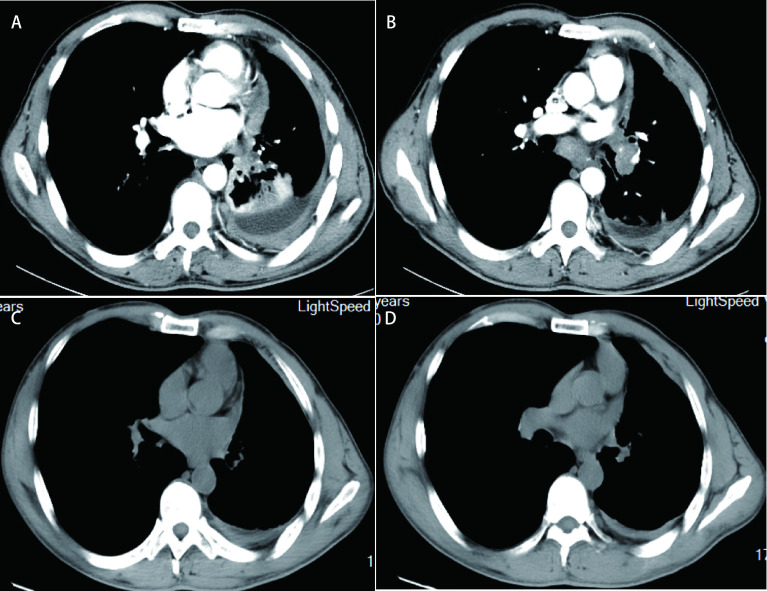
克唑替尼治疗前后CT图像。A：克唑替尼治疗前CT图像(2016年3月24日)：左侧包裹性胸腔积液; B：克唑替尼治疗前CT图像(2016年3月24日)：纵隔淋巴结肿大，左肺下叶占位(2.6 cm×1.4 cm); C：克唑替尼治疗后CT图像(2019年6月25日)：左侧包裹性胸腔积液较前减少; D：克唑替尼治疗后CT图像(2019年6月25日)：纵隔淋巴结肿大较前缩小，左肺下叶小病灶(1.3 cm×0.6 cm)缩小。 CT images before and after crizotinib treatment. A: CT images before crizotinib treatment (24-Mar-2016): left encapsulated pleural effusion; B: CT images before crizotinib treatment (24-Mar-2016): mediastinal lymphadenopathy, left lung lower lobe space occupying (2.6 cm×1.4 cm); C: CT images after crizotinib treatment (25-Jun-2019): left encapsulated pleural effusion was reduced; D: CT images after crizotinib treatment (25-Jun-2019): mediastinal lymph node enlargement was reduced, small lesion in the lower lobe of the left lung (1.3 cm×0.6 cm) was reduced.

**图 3:**
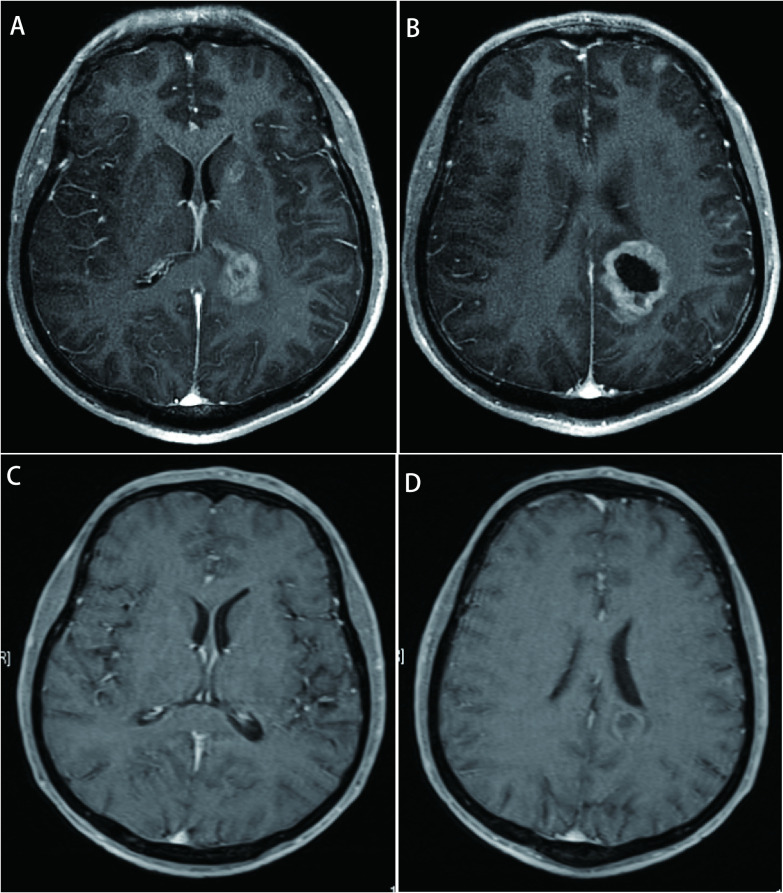
头MRI所示转移瘤情况。A：头MRI(2019年8月6日)：左侧基底节区，左侧脑室体旁转移瘤; B：头MRI(2019年8月6日)：左侧额叶皮层，左侧顶叶皮层，左侧脑室体旁转移瘤; C：头MRI(2020年1月20日)：左侧基底节区缩小，左侧脑室体旁转移瘤缩小; D：头MRI(2020年1月20日)：左侧额叶皮层，左侧顶叶皮层，左侧脑室体旁转移瘤缩小。 Brain MRI showing metastatic tumors. A: brain MRI (6-Aug-2019): metastatic tumor in left basal ganglia region, metastatic tumor near the body of the left lateral ventricle; B: brain MRI (6-Aug-2019): metastatic tumor in left frontal cortex, metastatic tumor in left parietal cortex, metastatic tumor near the body of the left lateral ventricle; C: brain MRI (20-Jan-2020): metastatic tumor in left basal ganglia region was reduced, metastatic tumor near the body of the left lateral ventricle was reduced; D: brain MRI (20-Jan-2020): metastatic tumor in left frontal cortex was reduced, metastatic tumor in left parietal cortex was reduced, metastatic tumor near the body of the left lateral ventricle was reduced. MRI: magnetic resonance imaging.

**图 4:**
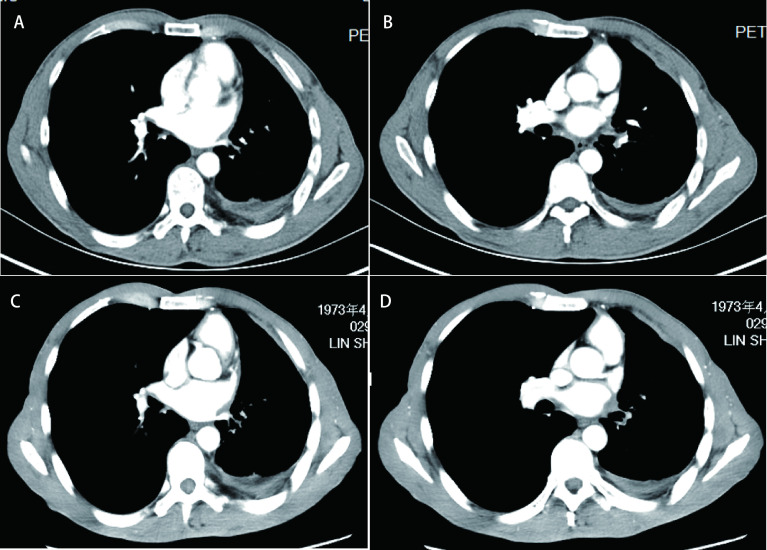
恩沙替尼治疗期间CT图像。A：CT(2019年11月13日)：左侧少量胸腔积液; B：CT(2019年11月13日)：纵隔淋巴结，左肺下叶小病灶; C：CT(2020年08月20日)：左侧胸腔积液同前; D：CT(2020年08月20日)：纵隔淋巴结同前，左肺下叶病灶同前。 CT images during treatment with ensartinib. A: CT (13-Nov-2019): a small pleural effusion on the left; B: CT (13-Nov-2019): mediastinal lymph node, small lesion in the lower lobe of the left lung; C: CT (20-Aug-2020): the small pleural effusion on the left was as same as before; D: CT (20-Aug-2020): the mediastinal lymph node was as same as before, the small lesion in the lower lobe of the left lung was as same as before.

## 2 讨论

2007年*ALK*基因被发现为肺癌治疗的潜在靶点^[1]^，作为继*EGFR*第二常见的驱动基因变异，ALK相关靶向药物的研发也异常迅猛。2014年PROFILE1014研究^[2]^提示，克唑替尼组对比化疗组中位PFS为10.9个月*vs* 7.0个月，克唑替尼组显出明显优势，该研究奠定了克唑替尼治疗ALK融合突变阳性肺癌患者的一线治疗地位，后续二代、三代ALK-TKI的出现，使大多数*ALK*融合突变阳性的肺癌患者在生存质量及生存期方面得到了很大改善^[2-6]^。前期曾有病例报道，经过治疗的ALK阳性患者总生存期能够超过7年，比如法国的IFCT-1302 CLINALK研究^[7]^中患者的中位OS为89.6个月; 日本WJOG9516L研究^[8]^中克唑替尼序贯阿来替尼中位OS达到88.44个月。此外一项来自美国UCCC的回顾性研究^[9]^分析提示，一线克唑替尼序贯后代ALK-TKI治疗患者的中位OS达到了86个月。随着ALK-TKI如雨后春笋般推陈出新，克唑替尼一线治疗的中位PFS仍显不够长，通常治疗10个月后，50%的患者会出现耐药，治疗12个月后，41.4%的患者可能发生脑转移^[10]^。在2019年欧洲肿瘤内科学会(European Society for Medical Oncology, ESMO)年会上，阿来替尼组中位PFS定格在34.8个月，对于基线无脑转移的患者，中位PFS更是高达38.6个月。如今，美国国立综合癌症网络(National Comprehensive Cancer Network, NCCN)指南、中国临床肿瘤学会(Chinese Society of Clinical Oncology, CSCO)肺癌指南已将阿来替尼推荐至一线治疗，而且为优先选择^[11, 12]^。

对于患者在一线全身治疗期间发现*ALK*重排，NCCN建议可选择完成化疗计划(包括维持治疗)，亦可选择中断化疗，直接给予阿来替尼、布加替尼、色瑞替尼或克唑替尼治疗^[13]^。2014年及2018年PROFILE1014研究中克唑替尼组和化疗组总生存期未见显著的统计学差异。一线应用ALK-TKI确实在PFS方面有明显获益，但OS获益并不明确，这可能也是NCCN指南并未摒弃一线应用化疗的原因之一。

病例报道中患者三线应用克唑替尼PFS较长。2016年的一项荟萃分析^[14]^检索了多个数据库涵盖约6, 086篇文献，意在对比在一线及二线应用克唑替尼的获益情况，该分析提示对于晚期*ALK*阳性NSCLC患者，一线应用克唑替尼可能比二线应用克唑替尼更获益(PFS为11.28个月*vs* 8.12个月)，而三线及后线应用克唑替尼的文献报道及数据较少。该患者前期因经济原因未能一线直接加用ALK-TKI治疗，先给予紫杉醇+顺铂方案化疗，后调整为培美曲塞+卡铂方案化疗，疗效达到PR且在维持PR的情况下改为克唑替尼治疗，三线应用克唑替尼PFS达到40个月，远远超出PROFILE1014研究中克唑替尼一线治疗中位PFS的10.9个月^[2]^。关于影响克唑替尼PFS的因素，前期曾有回顾性研究提示*ALK*融合阳性患者先应用含培美曲塞方案化疗，后行ALK-TKI治疗后可获得更久的PFS^[15, 16]^，而应用不包含培美曲塞的含铂类方案化疗，后续给予ALK-TKI治疗，PFS却并无优势^[16-18]^。这也许可以作为该患者PFS延长原因的探讨方向。*ALK*融合突变分为7个亚型，分别为V1、V2、V3a/b、V5a/b、V5’、V7以及Non-EML4-ALK亚型。其中V1、V2、V3占比较高，分别为43%、6%及40%^[19]^。2016年韩国的一项回顾性研究^[20]^提示，一线应用含有培美曲塞的方案化疗，*ALK*阳性患者PFS更具优势，进一步细分融合突变亚型，V1类型则表现出更优异的PFS。这一亚型是*ALK*融合突变类型占比最多的亚型。该研究中的多变量分析证明分型为V1类型是一线应用培美曲塞治疗延长PFS的唯一显著预测因素。值得注意的是，V1亚型同样是应用克唑替尼效果较好、继发耐药概率(尤其是继发*ALK* G1202R突变概率)较低的亚型^[19, 21]^。2017年我国一项回顾性研究提示，ALK阳性肺腺癌患者一线应用含培美曲塞方案化疗，客观缓解率(objective response rate, ORR)和疾病控制率(disease control rate, DCR)均高于阴性患者^[22]^，同样提示培美曲塞本身对*ALK*阳性患者更敏感。该患者三线应用克唑替尼的PFS相对较长，具体原因及机制有待进一步基础及临床研究的验证。

近些年来，中国原研ALK-TKI恩沙替尼研究数据不断发布，为*ALK*阳性NSCLC的治疗带来新的选择。恩沙替尼对*ALK*野生型及17个*ALK*突变型的抑制图谱分析结果显示，该药对所有*ALK*突变型均有明显的抑制作用，半抑制浓度(half maximal inhibitory concentration, IC_50_) < 4 nmol/L，其中对野生型*ALK*融合和继发F1174、C1156Y、L1196M、S1206R、T1151等突变位点显示出了强烈的抑制作用，IC_50_均小于0.4 nmol/L; 而对G1202突变体的抑制能力则相对较弱，IC_50_值为3.8 nmol/L^[23, 24]^。在前期报道的I期/II期研究中，恩沙替尼在*ALK*阳性患者中整体ORR为60%，DCR为81.7%，其中14例基线合并脑转移患者ORR为64.3%，DCR为92.9%，提示该药对合并脑转移的患者可能更敏感。恩沙替尼主要不良反应为皮疹(56%)、恶心(36%)、瘙痒(28%)、呕吐(26%)和疲劳(22%)^[23]^。2019年10月关于恩沙替尼II期研究^[25]^数据再次肯定了恩沙替尼的安全性及效果，提示恩沙替尼可能成为潜在的一线治疗方案选择。相信不久的将来会有更多的数据更新为我们带来惊喜。
